# cAMP-independent DNA binding of the CRP family protein DdrI from *Deinococcus radiodurans*

**DOI:** 10.1128/mbio.01144-24

**Published:** 2024-06-25

**Authors:** Yudong Wang, Jing Hu, Xufan Gao, Yuchen Cao, Shumai Ye, Cheng Chen, Liangyan Wang, Hong Xu, Miao Guo, Dong Zhang, Ruhong Zhou, Yuejin Hua, Ye Zhao

**Affiliations:** 1 MOE Key Laboratory of Biosystems Homeostasis & Protection, Institute of Biophysics, College of Life Sciences, Zhejiang University, Hangzhou, China; 2 Institute of Quantitative Biology, College of Life Sciences, Zhejiang University, Hangzhou, China; 3 Shanghai Institute for Advanced Study, Zhejiang University, Shanghai, China; 4 Department of Chemistry, Columbia University, New York, New York, USA; The Pennsylvania State University, University Park, Pennsylvania, USA

**Keywords:** transcription factor, DNA binding, dimer, cAMP, allosteric effect, microscale thermophoresis

## Abstract

The cAMP receptor proteins (CRPs) play a critical role in bacterial environmental adaptation by regulating global gene expression levels via cAMP binding. Here, we report the structure of DdrI, a CRP family protein from *Deinococcus radiodurans*. Combined with biochemical, kinetic, and molecular dynamics simulations analyses, our results indicate that DdrI adopts a DNA-binding conformation in the absence of cAMP and can form stable complexes with the target DNA sequence of classical CRPs. Further analysis revealed that the high-affinity cAMP binding pocket of DdrI is partially filled with Tyr113-Arg55-Glu65 sidechains, mimicking the *anti*-cAMP-mediated allosteric transition. Moreover, the second *syn*-cAMP binding site of DdrI at the protein-DNA interface is more negatively charged compared to that of classical CRPs, and manganese ions can enhance its DNA binding affinity. DdrI can also bind to a target sequence that mimics another transcription factor, DdrO, suggesting potential cross-talk between these two transcription factors. These findings reveal a class of CRPs that are independent of cAMP activation and provide valuable insights into the environmental adaptation mechanisms of *D. radiodurans*.

**IMPORTANCE:**

Bacteria need to respond to environmental changes at the gene transcriptional level, which is critical for their evolution, virulence, and industrial applications. The cAMP receptor protein (CRP) of *Escherichia coli* (ecCRP) senses changes in intracellular cAMP levels and is a classic example of allosteric effects in textbooks. However, the structures and biochemical activities of CRPs are not generally conserved and there exist different mechanisms. In this study, we found that the proposed CRP from *Deinococcus radiodurans*, DdrI, exhibited DNA binding ability independent of cAMP binding and adopted an apo structure resembling the activated CRP. Manganese can enhance the DNA binding of DdrI while allowing some degree of freedom for its target sequence. These results suggest that CRPs can evolve to become a class of cAMP-independent global regulators, enabling bacteria to adapt to different environments according to their characteristics. The first-discovered CRP family member, ecCRP (or CAP) may well not be typical of the family and be very different to the ancestral CRP-family transcription factor.

## INTRODUCTION

Glucose is the preferred carbon source in *Escherichia coli*. Early studies on *E. coli* lacking glucose during growth led to the discovery of elevated levels of the intracellular signaling molecule cyclic 3ʹ,5ʹ-AMP (cAMP), which activates the DNA binding of the cAMP receptor protein (CRP) and regulates global gene expression of more than 7% of genes. This phenomenon is known as catabolite repression, enabling bacteria to utilize non-preferred carbon sources such as lactose (1, 2). Later, bacterial CRPs have been shown to be involved in a wider range of regulatory effects related to environmental adaptation, such as quorum sensing (3), iron acquisition (4), virulence (5), and motility (6). Moreover, CRPs may also play a non-transcriptional regulatory role in some bacteria (7, 8). For example, cAMP-CRP is hypothesized to be required for *Shewanella putrefaciens* biofilm maintenance through physical interaction with BpfD (8).

*E. coli* CRP (ecCRP) was the first transcription factor with a crystal structure available and serves as a model for biochemical and structural analyses (9–11). ecCRP exists as a dimer with each protomer containing an N-terminal domain and a C-terminal domain. These two domains are linked by a long helix (C-helix) forming a hydrophobic dimerization interface. While the primary cAMP binding pocket (dissociation constant (*K*_d_) of about 27 µM) is located in the N-terminal domain, the C-terminal domain of ecCRP contains a helix-turn-helix motif responsible for DNA binding. Interestingly, a second cAMP binding site with much lower affinity (*K*_d_ ≈ 2 mM) was observed between these two domains. It is worth noting that the primary cAMP binding pocket is far away from the helix-turn-helix motif, suggesting an allosteric regulation mechanism. The binding of cAMP results in rearrangements of C-helix (coil-to-helix transition), D-helix, and β4-β5 loop of ecCRP, further reorientating the F-helix in a position optimal for DNA major groove binding. Biochemical and structural studies revealed that ecCRP can bind palindrome consensus sequences, which sharply kinks the DNA duplex to varying degrees (12, 13). It has also been proposed that the cAMP binding shifts the equilibrium of inactive CRP toward the active form, which explains the unusual cAMP binding affinity of CRP mutants (CRP*) (11, 14). Interestingly, CRPs from some bacteria are not sensitive to cAMP binding; for example, the DNA binding affinity of *Mycobacterium tuberculosis* CRP (mtCRP) changes little upon cAMP binding (15, 16).

*Deinococcus radiodurans* is one of the important model organisms for studying the environmental adaptability mechanisms of bacteria. It can survive under extreme environmental stresses such as high-dose gamma radiation (*D*_10_ value of 10 kGy), DNA damage reagents (e.g., mitomycin C), and oxidative stress treatments, due to its super DNA damage repair ability and antioxidant capacity (17–19). However, how this radiation resistance evolved remains an open-ended question. One attractive theory is that *D. radiodurans* originated on Earth and specifically evolved super DNA damage repair ability in a long term drought environment. Its extreme ionizing radiation resistance is a by-product of adaptation to drought stress. Indeed, in addition to its super radiation-resistance, *D. radiodurans* can survive for a long-time during desiccation, which can also cause numerous DNA double-strand breaks in cells (similar to the biological effects of high-dose ionizing radiation). Transcriptome analysis further provides direct evidence for this hypothesis: a considerable part of the upregulated genes in response to drought and radiation stress in *D. radiodurans* overlap, and there is a palindromic consensus sequence in the promoter region of these genes, named radiation/desiccation response motif (RDRM) (20–22). These RDRM-containing genes are not only involved in the classical DNA repair processes (e.g., *recA* and *ssb*, which are also found in other bacteria) but also Deinococcus-specific genes with novel functions, such as some *ddr* (DNA damage response) series genes (23–25). Recent studies have uncovered an elegant RDRM regulation mechanism by a transcription factor protein DdrO and metalloprotease PprI (26–28).

*D. radiodurans* contains four putative CRP family proteins, including DR0997 (also known as DdrI), DR1646, DR2362, and DR0834. Among these candidates, DdrI was identified as playing a major role in global regulation for the adaptation of *D. radiodurans* to various stresses (29, 30). *ddrI* was highly induced by gamma radiation treatments, and the inactivation of this gene sensitized cells to various stress environments including heat shock, radiation, DNA-damaging agents, and oxidation. Efforts to characterize the DdrI binding sequence using electrophoretic mobility shift assay (EMSA) and *in silico* methods have led to the identification of hundreds of potential DdrI target genes involved in divergent functions in metabolic pathways. Moreover, DdrI was also shown to be involved in cell division, genome segregation, and plasmid maintenance (29). It should be noted that *ddrI* does not belong to the RDRM gene group, and ecCRP only partially rescued the phenotype of the *ddrI* knockout strain.

In this work, we have determined the crystal structure of *D. radiodurans* DdrI, which forms a dimer via coiled-coil interactions by fully folded C-helix of two neighboring protomers in the absence of cAMP. Together with the properly positioned HTH motif for binding to DNA, this DdrI structure resembles the cAMP-activated CRP structure. Biochemical analysis suggested that DdrI exhibited a very low affinity for cAMP binding. The addition of cAMP can, to a certain extent, decrease the affinity of DdrI for target DNA. In contrast, Mn^2+^ was able to enhance DdrI binding to target DNA, especially sequences containing the RDRM-mimicking variant, indicating potential cross-talk between different transcription factors in *D. radiodurans* in response to environmental stress.

## RESULTS

### Overview of the DdrI dimer structure

**Fig 1:**
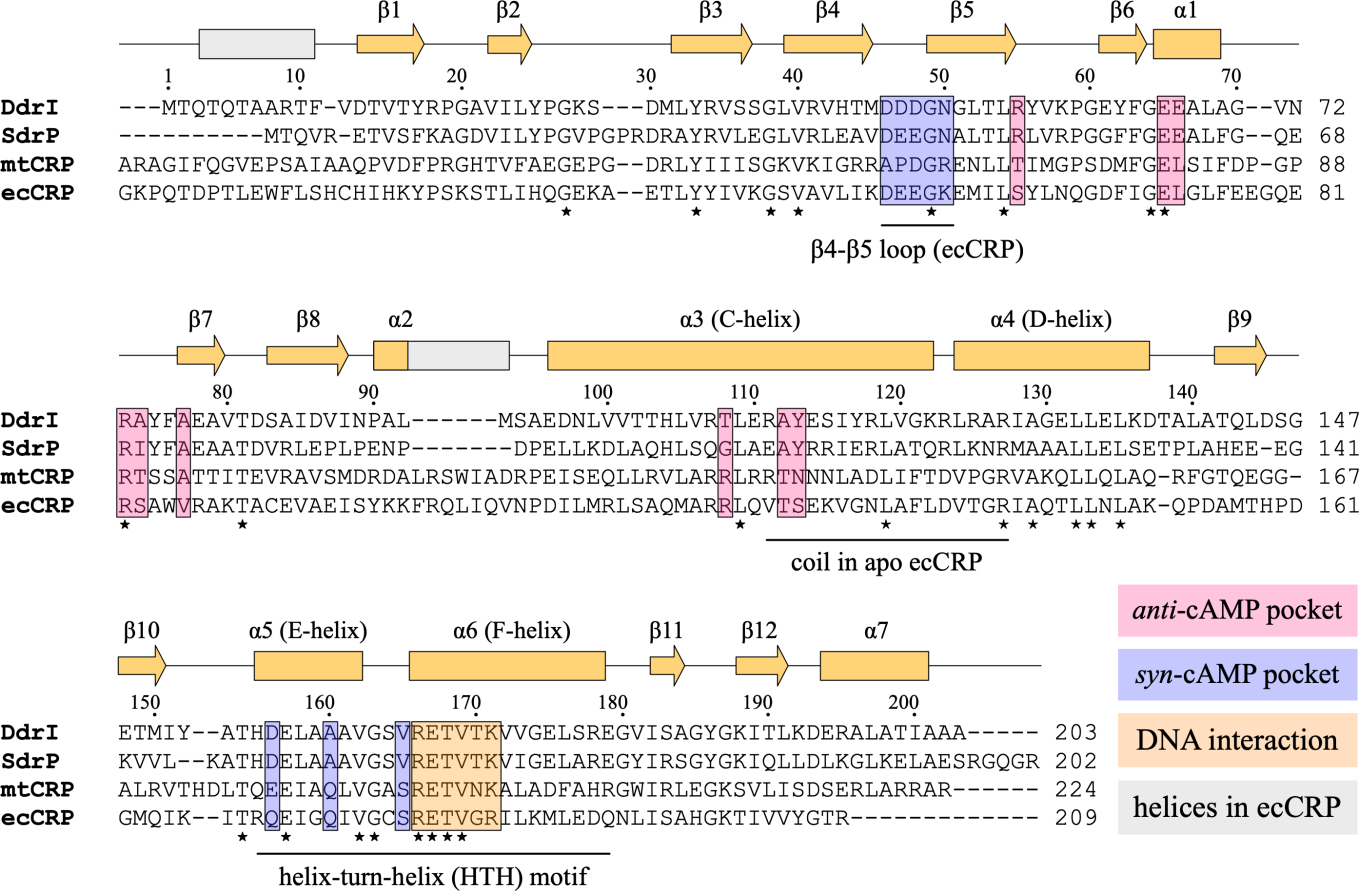
Structure-based sequence alignment of CRPs from *Deinococcus radiodurans* (DdrI), *Thermus thermophilus* (SdrP), *Mycobacterium tuberculosis* CRP (mtCRP), and *Escherichia coli* (ecCRP). Secondary structures of DdrI (orange) and ecCRP (gray) are shown on top. Conserved residues are indicated by stars below the alignment. Residues involved in *anti*-cAMP binding pocket, *syn*-cAMP binding site, and DNA interactions are boxed and highlighted in distinct colors.

As noted in a previous study (30), *D. radiodurans* DdrI contains 203 amino acids, which is 57 amino acids shorter than the original annotation. In addition to being conserved within Deinococcus species, DdrI can be aligned with CRPs from other bacteria, sharing 52%, 29%, and 27% amino acid identities with its homologs from *Thermus thermophilus* (SdrP), *M. tuberculosis* (mtCRP), and *E. coli* (ecCRP), respectively (Fig. 1). DdrI has a calculated isoelectric point of 5.30, which is similar to that of SdrP (5.43) but significantly lower than that of ecCRP (8.38) and that of mtCRP (9.57).

**Fig 2:**
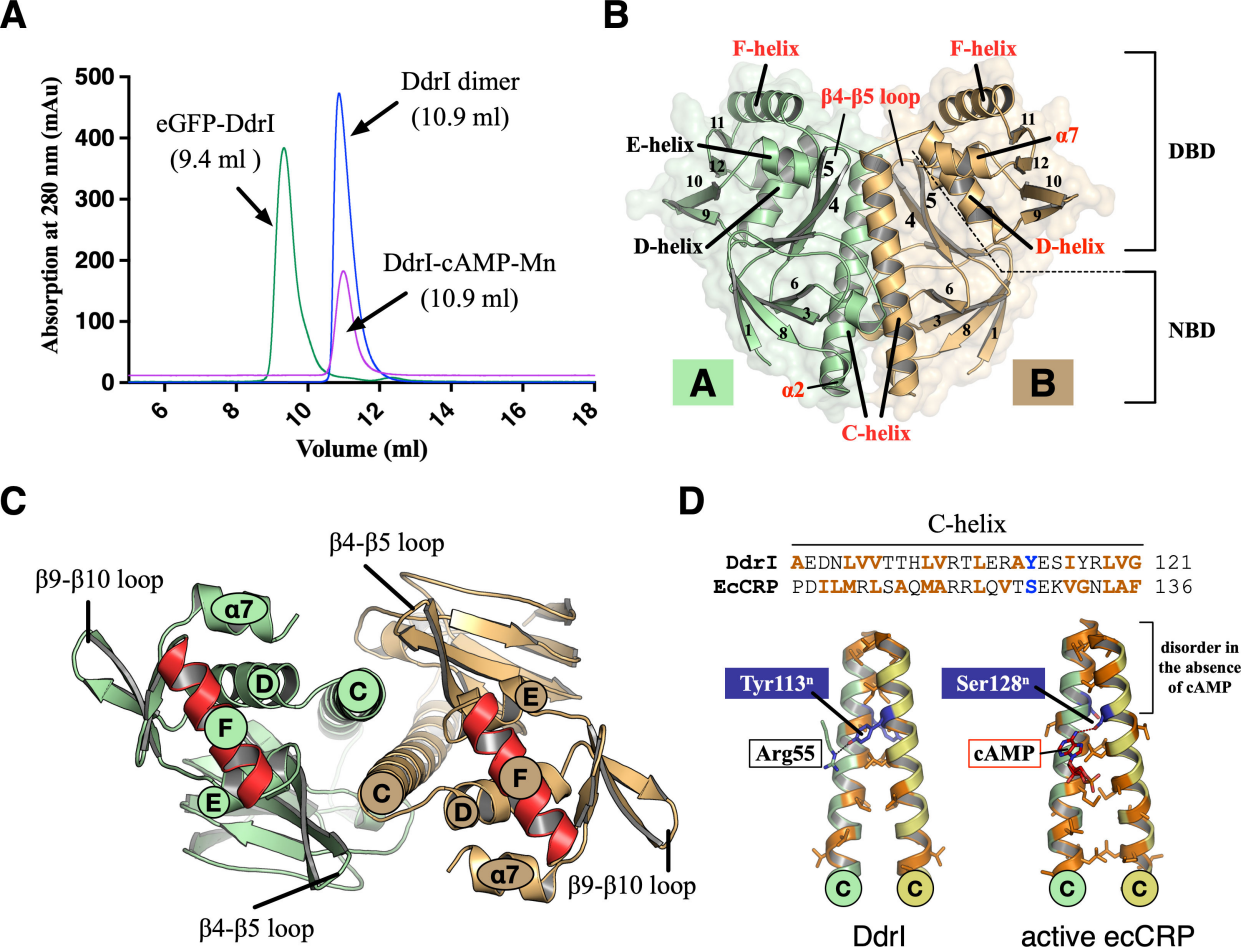
Overview of the DdrI dimer structure. (**A**) Size-exclusion chromatography demonstrates the presence of dimeric DdrI and eGFP-DdrI. The addition of cAMP and Mn^2+^ (DdrI-cAMP-Mn) did not affect its dimer formation. (**B**) Side view of the overall structure of DdrI. The DdrI dimer is depicted in cartoon representation, with two protomers colored green (**A**) and wheat (**B**), respectively. The α-helices, β-strands, nucleotide-binding N-terminal domain (NBD), and DNA-binding C-terminal domain (DBD) of each protomer are labeled. (**C**) Top view of the overall structure of the DdrI dimer. Functionally important α-helices and loops are labeled, with the F-helix highlighted in red. (**D**) The C-helices involved in CRP dimerization. The amino acid sequence is shown on top, with hydrophobic residues in orange. cAMP (shown in red) and the hydrophobic residues (orange sticks) are displayed, with Tyr113 (DdrI) and Ser128 (ecCRP) highlighted in blue.

Full-length DdrI containing a fused N-terminal 6 × His tag, an MBP-tag, and a TEV protease recognition sequence was expressed and purified. After tag removal during the purification steps, the untagged DdrI was subjected to gel filtration chromatography to check its homogeneity and oligomeric properties. Unsurprisingly, DdrI exists as a homodimer in solution (43.9 kDa, Fig. 2A). DdrI crystals were grown and diffracted X-rays to 2.0 Å (Table S1). While the asymmetric unit contains one DdrI monomer, two neighboring molecules form a dimer (Fig. 2B), consistent with its dimerization in solution (Fig. 2A). The final model, which comprises 7 α-helices and 12 β-strands, contains two domains: a nucleotide-binding N-terminal domain (NDB, residues 13–94) and a DNA-binding C-terminal domain (DBD, residues 122–202). These two domains are interconnected by a long linker α-helix (C-helix; residues 95–121). The NDB of DdrI shares a canonical jelly roll topology with two layers of antiparallel β-strands, and DBD contains a winged HTH motif (E- and F-helices) critical for DNA binding (Fig. 2B and C). We performed a search with previously determined CRP structures in the PDB database (DALI server) using our DdrI structure as the query, resulting in the closest structure of SdrP, the CRP family protein from *T. thermophilus* (PDB ID: 2ZCW), with root mean square deviation (RMSD) of 1.2 Å over 188 pairs of Cα atoms. As a member of the CRP family proteins, the DdrI structure can also be aligned with other CRPs in their apo form, including ecCRP (PDB ID: 2WC2, RMSD value of 3.2 Å over 183 pairs of Cα atoms), and mtCRP (PDB ID: 3D0S, RMSD value of 2.1 Å over 189 pairs of Cα atoms).

Compared with ecCRP, the DdrI structure exhibits variations in secondary structures (Fig. 1 and 2B). The very first N-terminal α-helix observed in ecCRP is disordered in the DdrI structure. The α2 helix of DdrI preceding the C-helix contains only three amino acids, which is much shorter than that of ecCRP (10 residues). An additional α-helix (α7) at the C-terminus of the DBD of DdrI packs with the D-helix (Fig. 2C), which is not present in ecCRP. Importantly, the linker C-helix in the DdrI structure is fully folded, in contrast to the partial folding of this region in ecCRP in the absence of cAMP (Fig. S1A and S1B; Fig. 2D) (31).

### The apo structure of DdrI resembles the active CRP form

**Fig 3:**
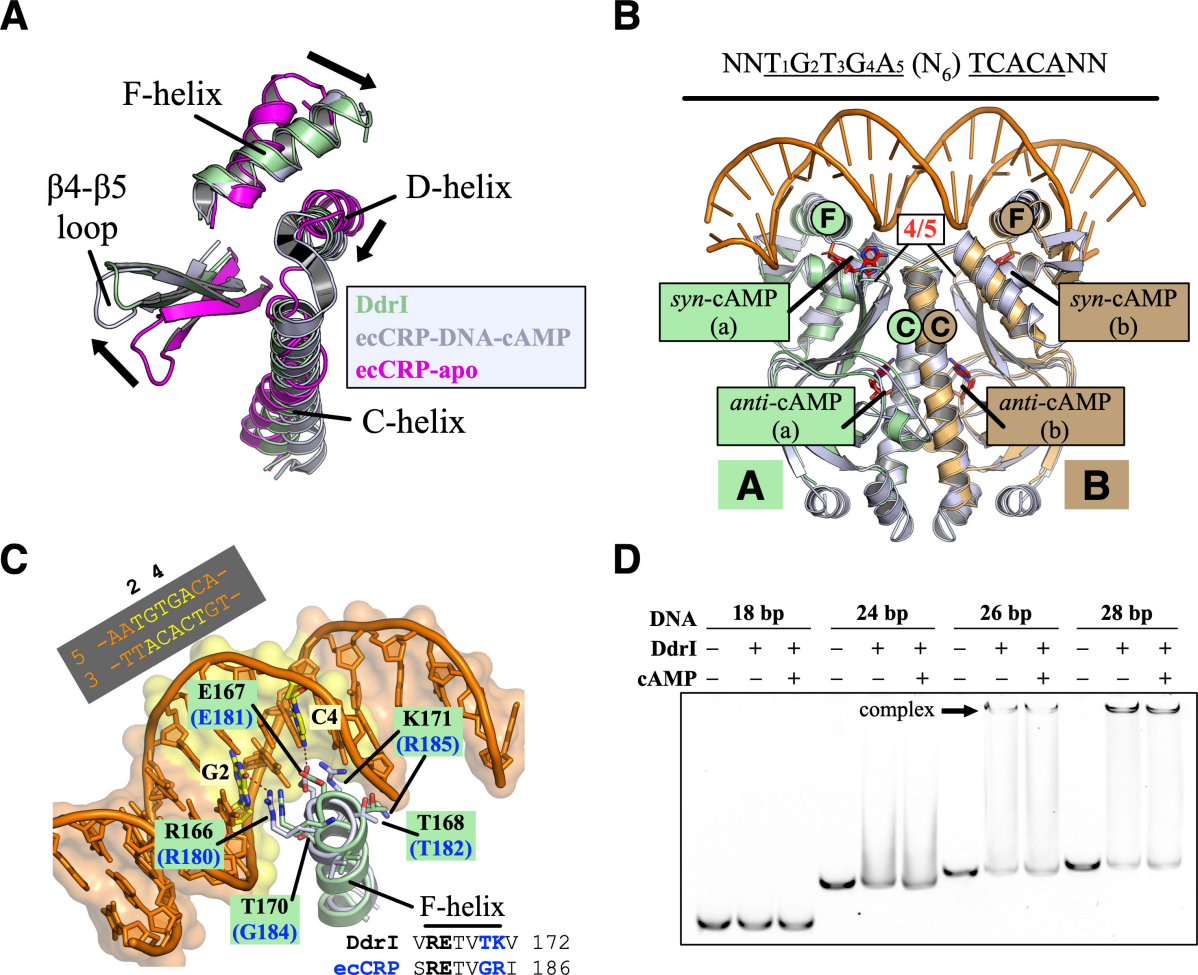
Apo DdrI structure resembles the active CRP form. (**A**) Superposition of the secondary elements critical for allosteric transition of CRP. The structures of DdrI (from this study), apo ecCRP (PDB ID: 2WC2), and ecCRP-DNA-cAMP (PDB ID: 2CGP) are colored green, magenta, and gray, respectively. The black arrowheads indicate the shifts in the C-helix (coil-to-helix transition), D-helix, F-helix, and β4-β5 loop that occur during the activation of ecCRP by cAMP. (**B**) Superposition of the DdrI dimer (colored as in Fig. 2B) with the ecCRP-DNA-cAMP complex (gray). Two cAMP binding sites from each protomer are labeled, with the cAMP molecules shown as red sticks. The C-helix, F-helix, and β4-β5 loop (4/5) are labeled. (**C**) The zoom-in view of the F-helices of DdrI (green) and ecCRP-DNA-cAMP (gray), with the palindrome consensus sequence indicated above. The F-helix residues of DdrI (labeled in black) and ecCRP (labeled in blue) are shown as sticks. The half-site of the palindrome consensus sequence is highlighted in yellow, with G_2_ and C_4_ labeled. The red dashed lines indicate the interactions between protein and DNA. (**D**) EMSA shows the DNA binding capability of DdrI. 5′-Cy5-labeled target DNA containing the palindrome consensus sequence (100 nM, with lengths of 18–28 bp) was incubated with 1 µM DdrI protein in the absence or presence of 100 µM cAMP at 30°C for 30 min. Black arrowheads indicate the stable complexes.

The coil-to-helix transition of the C-helix is the key structural feature distinguishing between active and inactive ecCRP structures (10, 11). Thus, the fully folded C-helix of apo DdrI led us to suspect that our DdrI without cAMP binding represents the active CRP form. Indeed, the superposition of the DdrI dimer with the ecCRP-DNA-cAMP complex (PDB ID: 2CGP) results in an RMSD value of 2.1 Å over 181 pairs of Cα atoms (Fig. S1A). Despite the slight movement of solvent-exposed loops, two cAMP binding sites and the HTH motif of DdrI could align well with those in ecCRP-DNA-cAMP structure. Notably, compared with ecCRP apo structure lacking the cAMP, the β4-β5 loop and D-helix show noticeable movement, with the F-helix being in a position compatible with target DNA binding (Fig. 3A).

Given the good superimposition between DdrI and ecCRP-DNA-cAMP structure, target DNA containing TGTGA(N6)TCACA could easily be docked onto the HTH motif of DdrI (Fig. 3B). Previous studies have suggested that ecCRP protomer recognizes target DNA through interactions between the DNA half-site (T_1_G_2_T_3_G_4_A_5_) and the R_180_ETVGR_185_ motif of the F-helix, and the same on the other side. These F-helix residues are important for DNA binding affinity and specificity of ecCRP (Fig. 3C). While Arg180 forms a hydrogen bond with the guanine base of G_2_, the G_4_:C_4_ base pair is recognized by interactions between Arg185 and Glu181 with the guanine base (G_4_) and cytosine base (C_4_), respectively. Despite the conservation and similar rotamer states of the first four amino acids (R_166_ETV_169_), the last two amino acids of the DdrI F-helix, Thr170 and Lys171 (equivalent to Gly184 and Arg185 in ecCRP), are not conserved (Fig. 1 and 3C).

**Fig 4:**
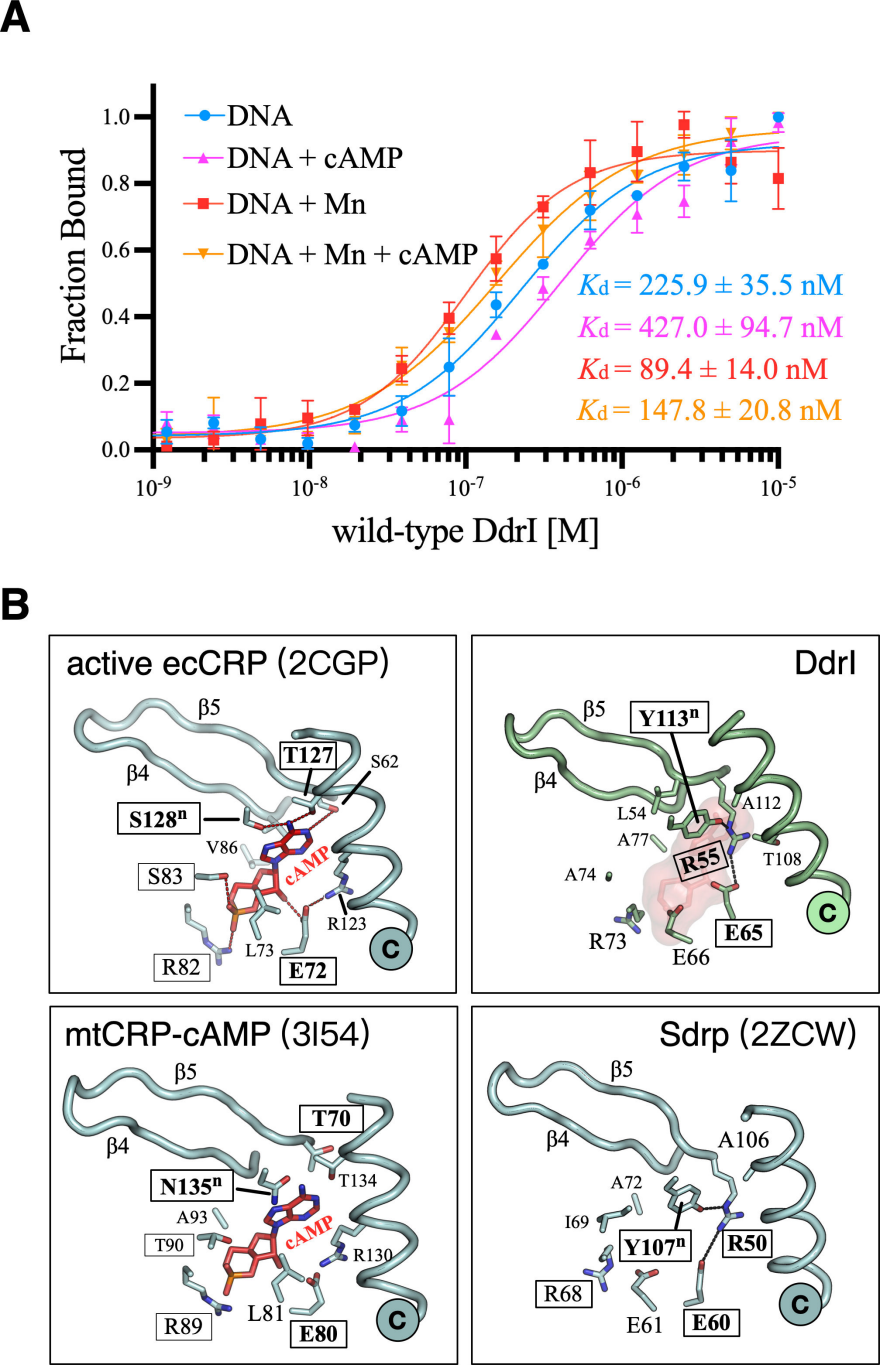
DNA binding affinity and the *anti*-cAMP binding site of DdrI. (**A**) Target DNA binding affinities determined by MST. Target DNA (28 bp) was labeled with 5′-Cy5 and maintained at a constant concentration of 20 nM in MST buffer at 30°C. This was followed by the titration of wild-type DdrI, ranging from 0.305 nM to 10 µM. If necessary, 10 µM Mn^2+^ and 100 µM cAMP were added. Binding curves represent the change in normalized fluorescence (Fraction Bound), and the *K*_d_ values were calculated using the K_d_ Fit Model. (**B**) The high-affinity cAMP (*anti*-cAMP) binding pocket of CRPs from ecCRP (PDB ID: 2CGP), DdrI (this study), mtCRP (PDB ID: 3I54), and SdrP (PDB ID: 2ZCW). The C-helix, β4, and β5 are shown as ribbons and labeled. The *anti*-cAMP and cAMP-interacting residues are shown as sticks and labeled. Residues equivalent to the TS motif (Thr127-Ser128), RS motif (Arg82-Ser83), and Glu72 of ecCRP are boxed. The dashed lines indicate the interactions between residues and cAMP within this binding pocket.

EMSA using different lengths of target DNA containing TGTGA(N6)TCACA was performed to further confirm the DNA binding affinity of DdrI in the absence of cAMP (Fig. 3D). DdrI was able to form a stable complex with target DNA in a length-dependent manner: target DNA of 26 bp constituted a length threshold, below which no stable complex was detected (Fig. 3D). The DNA binding affinity constant of DdrI was measured by microscale thermophoresis (MST) analysis. Target DNA (28 bp) was labeled with 5ʹ-Cy5 and kept at a constant concentration of 20 nM in MST buffer at 30°C, followed by DdrI titration from 0.305 nM to 10 µM. Fitting the data according to the K_d_ Fit Model resulted in a dissociation constant of *K*_d_ = 225.9 ± 35.5 nM (Fig. 4A; Table 1). These results indicate that DdrI can form an active dimer in the absence of cAMP molecules, which is consistent with structural observations (Fig. 3A and B).

**TABLE 1:** DNA binding affinities determined by MST

DNA	Protein	Mn^2+^	cAMP	*K*_d_ (nM)
Target DNA	WT	−	−	225.9 ± 35.5
		−	+	427.0 ± 94.7
		+	−	89.4 ± 14.0
		+	+	147.8 ± 20.8
	D156Q	−	−	45.9 ± 8.5
		−	+	32.7 ± 7.7
		+	−	22.7 ± 3.4
		+	+	24.4 ± 4.6
*ddrB*-mimic	WT	−	−	No binding
		+	−	20.3 ± 6.4 µM

### The discrepancies in the high-affinity cAMP (*anti*-cAMP) binding pocket

The primary cAMP binding site is close to the coiled-coil dimerization interface formed by two neighboring C-helices (Fig. 3B). In the absence of cAMP, the C-terminal portion of the C-helix of apo ecCRP is unstructured (as loop region), interacting with the β4-β5 loop of the neighboring protomer (Fig. S1A). Upon the cAMP accommodation, β4 and β5 exhibit noticeable movement, resulting in the β4-β5 loop shifting toward the DBD (Fig. 3A). In addition, the C-helix directly interacts with the cAMP, undergoing a substantial coil-to-helix transition to be fully folded into a long α-helix. Not surprisingly, this cAMP binding pocket of DdrI appears to be well structured in terms of the arrangement of surrounding secondary structure elements (Fig. 3B and 4B).

There are discrepancies concerning the cAMP interacting residues based on structural-based sequence alignment (Fig. 1 and 4B). cAMP is bound in the *anti*-conformation in classic CRP complexes (e.g., ecCRP and mtCRP) through several key residues (Fig. 4B). In active ecCRP (PDB ID: 2CGP), the phosphoribose of cAMP is held in place by Glu72, Leu73, Arg82, Ser83, and Val86; the adenine base interacts with Ser62, Thr127, and Ser128^n^ (n: from the neighboring protomer). In addition, Arg123 forms a salt bridge with Glu72, which is important for cAMP discrimination. Among these phosphoribose-interacting residues, DdrI has two conserved residues, Glu65 and Arg73 (equivalent to ecCRP Glu72 and Arg82) but three substitutions: Glu66, Ala74, and Ala77 (equivalent to ecCRP Leu73, Ser83, and Val86, respectively) (Fig. 1 and 4B). Notably, all the residues interacting with the adenine base are not conserved compared with ecCRP or mtCRP; they are replaced by Arg55, Thr108, Ala112, and Tyr113^n^ (equivalent to ecCRP Ser62, Arg123, Thr127, and Ser128^n^, respectively).

In contrast to the cavity occupied by the cAMP molecule in ecCRP, this binding pocket of DdrI is partially filled with Tyr113^n^-Arg55-Glu65 (equivalent to ecCRP Ser128^n^, Ser62, and Glu72) (Fig. 4B). Instead of interacting with the adenine base as its ecCRP equivalent does, Arg55 of DdrI forms hydrogen bonds with both Tyr113^n^ and Glu65, leading to insufficient space for cAMP accommodation. Moreover, Tyr113^n^ packs behind a leucine residue. This residue, Leu54, is conserved within CRPs, which participates in the dimerization interface formation through hydrophobic interactions in both DdrI and ecCRP structures. The addition of cAMP (100 µM) slightly decreased the DNA binding affinity of DdrI (Fig. 4A, DNA + cAMP). Thereby, putative cAMP binding to this site should be excluded.

### The second cAMP (*syn*-cAMP) binding site

The second cAMP molecule is bound in the *syn* conformation. This was first observed in the ecCRP-DNA structure in the presence of 2 mM cAMP during the crystallization (12). In contrast to the buried cAMP in the first binding pocket, this cAMP binding site is solvent-exposed. Given that this *syn*-cAMP is located between the DBD and NTD domains and interacts with the β4-β5 loop, HTH motif, neighboring C-helix, and DNA (Fig. 3B; Fig. S1A), it has been proposed to be involved in the activation of ecCRP by cAMP. The putative binding site of this *syn*-cAMP is also solvent-accessible in the DdrI structure.

**Fig 5:**
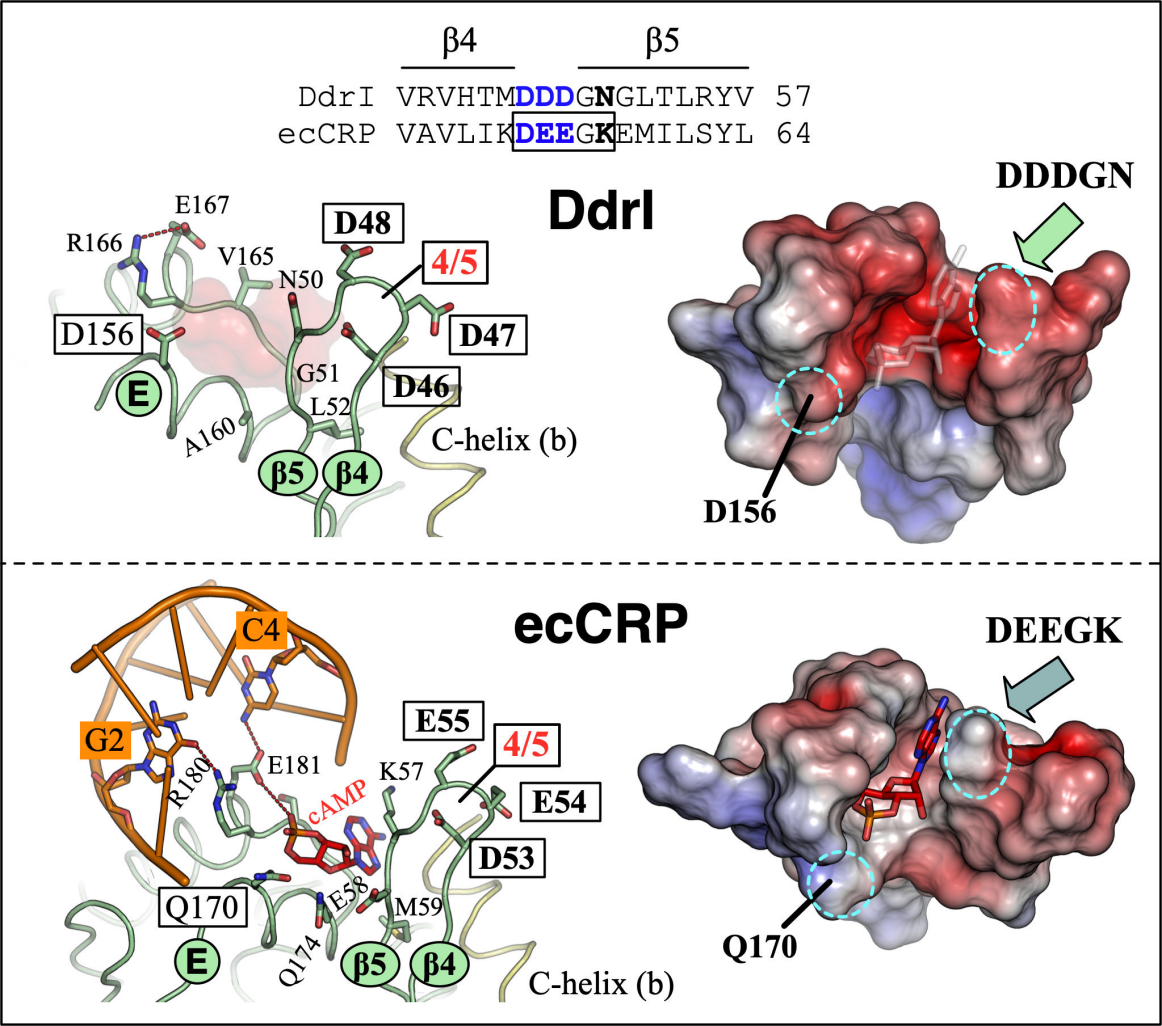
The second cAMP (*syn*-cAMP) binding site. Helices, β-strands, and β4-β5 loop (labeled 4/5) of DdrI (top) and ecCRP (PDB ID: 2CGP, bottom) are shown in ribbon representation with labels. Residues and *syn*-cAMP are depicted as sticks, colored green and red, respectively. The electrostatic potential of the *syn*-cAMP binding surface was determined using the APBS and is shown as a solvent-excluded surface. Blue and red denote positive and negative charge potentials, respectively, on a scale of ±7kTe^−1^. Dashed lines highlight interactions between residues and *syn*-cAMP within this binding pocket.

As a hydrophobic molecule, the adenosine of *syn*-cAMP in ecCRP-DNA complex packs against the C-terminus of the β4-β5 loop (Lys57-Glu58-Met59), Gln170, and Gln174, with the phosphate oxygen interacting with Arg180 (main chain) and Glu181 (a water-mediated interaction) (Fig. 5). The latter two amino acids are critical for the DNA binding of CRPs, interacting with the G-C base pairs at positions 2 and 4 of the target sequence (Fig. 3C). However, residues packing the putative *syn*-cAMP show deviations in DdrI, with Asn50-Gly51-Leu52, Asp156, and Ala160 in the corresponding positions (Fig. 5). Interestingly, variations of these β4-β5 loop residues lead to β-strand formation in this region, resulting in an elongated (two amino-acids longer) β5 strand compared with that of ecCRP. Moreover, the β4-β5 loop before these packing residues comprises triple-aspartate (Asp46-Asp47-Asp48) different from its equivalent in ecCRP (Asp46-Glu47-Glu48). Analysis of the distribution of the electrostatic surface potential suggested that this putative cAMP binding site, in addition to the β4-β5 loop, is more negatively charged in DdrI than in ecCRP (Fig. 5).

**Fig 6:**
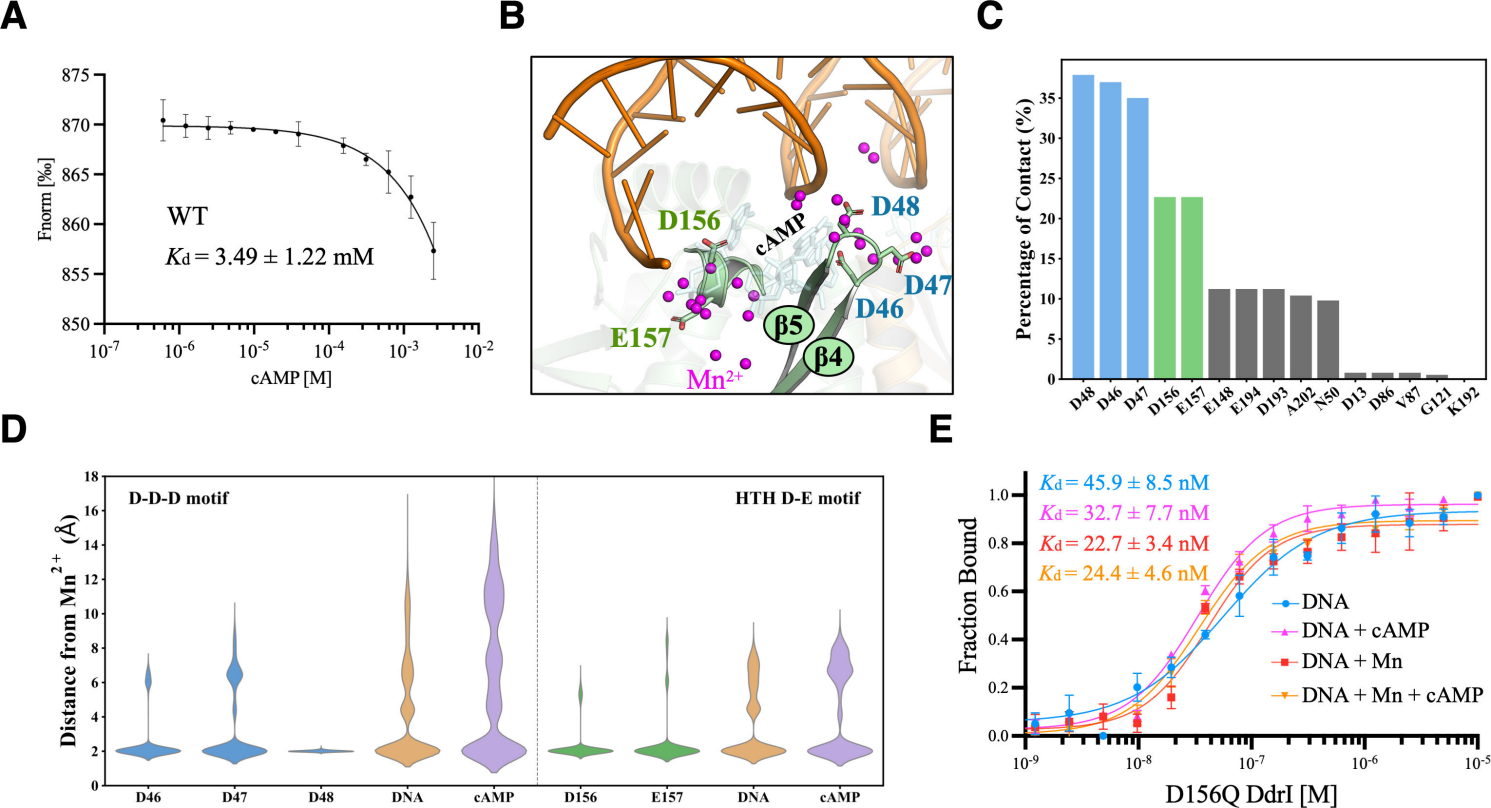
Molecular dynamics simulations reveal that Mn^2+^ mediates the interaction between DdrI and the RDRM-mimicking DNA. (**A**) The cAMP binding constant of wild-type DdrI. N-terminal fused eGFP-DdrI was kept at a constant concentration of 20 nM, followed by cAMP titration. Binding curves represent the change in normalized fluorescence (Fnorm[‰]), and the *K*_d_ values were calculated using the K_d_ Fit Model. (**B**) Molecular dynamics simulations of Mn^2+^ binding to DdrI in the presence of RDRM-mimicking DNA and cAMP. The E-helix, β4, and β5 strands are shown in cartoon representation with labels. Residues involved in two hotspots and Mn^2+^ are colored blue (Asp46-Asp47-Asp48), green (Asp156-Glu157), and magenta, respectively. (**C**) The percentage of contact with Mn^2+^ of all protein residues indicates predominant Mn^2+^ hotspots: Asp46-Asp47-Asp48 (blue) and Asp156-Glu157 (green). (**D**) Distribution of distances between Mn^2+^ and coordinating atoms on hotspot residues, DNA, and cAMP. (**E**) DNA binding affinities of D156Q mutant DdrI. Target DNA (28 bp) was labeled with 5′-Cy5 and maintained at a constant concentration of 20 nM in MST buffer at 30°C. This was followed by titration of D156Q mutant protein, ranging from 0.305 nM to 10 µM. If necessary, 10 µM Mn^2+^ and 100 µM cAMP were added. Binding curves represent the change in normalized fluorescence (Fraction Bound), and the *K*_d_ values were calculated using the K_d_ Fit Model.

Given the different local environment of the *syn*-cAMP binding site of DdrI, we checked the cAMP binding affinity by MST analysis (Fig. 6A). DdrI was expressed containing an N-terminal fused eGFP and kept at a constant concentration of 20 nM in MST buffer at 30℃. This was followed by cAMP titration from the nanomolar to the millimolar range. Low concentrations of cAMP (76.3 nM to 313.0 µM), corresponding to the concentrations used for high-affinity cAMP binding constants measurements of classical CRPs, showed no ligand binding. This was consistent with the EMSA assay and structural observations of the invalid *anti*-cAMP binding pocket of DdrI (Fig. 3D and 4B). Fitting the data according to the K_d_ Fit Model results in a dissociation constant of *K*_d_ = 3.49 ± 1.22 mM (Fig. 6A), a value in a range commensurate with *syn*-cAMP binding affinity of other CRPs (12).

### Enhancement of target DNA binding by Mn^2+^

Transcriptional regulation of gene expression at targets containing the RDRM motif is critical for the adaptation of *D. radiodurans* in response to environmental stresses (22). Based on the partial overlaps of the consensus sequences between DdrI regulons and the RDRM motif, it was hypothesized that DdrI may also play a regulatory role for some radiation/desiccation response (RDR) genes (29). Variant sequences containing a portion of RDRM (TGTTA, present in the RDRM sequences of *ddrB* and *ddrC*) were investigated for DdrI binding (Fig. S2). These variants contain a T-A base mutation at the G_4_:C_4_ position and various substitutions on the other half of DNA. Unlike the high-affinity binding of target DNA (Fig. 3D and 4A), DdrI was unable to bind these two variants under the same experimental conditions *in vitro*: no protein-DNA complex formation was observed by EMSA (Fig. S2A), and no binding signal of *ddrB*-mimic was detected by MST analysis (Fig. S2B).

*D. radiodurans* has an unusually high intracellular Mn concentration (millimolar), which can activate RDR gene expression through specific cleavage of DdrO by PprI protease (26, 27). Given that the *syn*-cAMP binding site of DdrI, which is close to the DNA binding surface, is more negatively charged (Fig. 5A), we were curious whether Mn also plays a role in DdrI regulation. The reactions containing Mn^2+^ resulted in dissociation constants of *K*_d_ = 89.4 ± 14.0 nM, which was approximately 2.5-fold lower than that without Mn^2+^ (Fig. 4A; Table 1). Interestingly, despite no stable complex being observed by EMSA assays, DdrI exhibited weak DNA binding capability to the *ddrB*-mimicking sequence (*K*_d_ = 20.3 ± 6.4 µM) in the presence of Mn^2+^ (Fig. S2B), which was two orders of magnitude higher than that of the target DNA. Interestingly, cAMP (100 µM) appeared to play a negative role in DdrI binding to DNA, resulting in approximate 1.9- and 1.6-fold increases in *K*_d_ values for target DNA binding in the absence and presence of Mn^2+^, respectively (Fig. 4A; Table 1).

Molecular dynamics simulations were performed to further investigate the possible role of Mn^2+^ involved in RDRM-mimicking DNA binding of DdrI. In the presence of Mn^2+^, cAMP appears to exhibit large fluctuation in the *syn*-cAMP binding site, partly exiting the vicinity of the β4 and β5 loops from time to time (Fig. 6B). Notably, Mn^2+^ predominantly congregates at two critical binding hotspots: (i) coordinated by Asp46, Asp47, and Asp48 of the β4-β5 loop; (ii) interacting with Asp156 and Glu157 (Fig. 6B and C). Interestingly, the findings in these two regions are consistent with the negative electrostatic potentials of the DdrI (Fig. 5). We next performed a second round of simulations to check the interactions between Mn^2+^ and surrounding residues. Mn^2+^ exhibits high specificity and stability to these residues, especially Asp48 and Asp156 (Fig. 6D). Moreover, in most replicas, the backbone phosphates of DNA and the cAMP are also involved in Mn^2+^ coordination (Fig. 6D); Mn^2+^ specifically neutralizes the negatively charged DNA-interacting interface of DdrI (Fig. 6B), facilitating more robust interactions between DdrI and DNA, thereby enhancing DdrI binding to the RDRM-mimicking DNA.

Asp156 was mutated to electrically neutral glutamine residue (D156Q, as its ecCRP equivalent) to measure its DNA binding affinities (Fig. 6E; Table 1). While the DNA binding constant for the D156Q mutant was *K*_d_ = 45.9 ± 8.5 nM, the addition of Mn^2+^ or Mn^2+^-cAMP resulted in increased target DNA binding (*K*_d_ = 22.7 ± 3.4 nM and *K*_d_ = 24.4 ± 4.6 nM, respectively). Moreover, cAMP was able to enhance the D156Q binding activity (*K*_d_ = 32.7 ± 7.7 nM). These numbers were approximately 10-fold lower than that of the wild-type DdrI, which are comparable to previous biochemical studies of activated mtCRP (15).

## DISCUSSION

Since the discovery of the very first CRP in *E. coli* more than five decades ago, the question of how transcription factors sense environmental stresses and transmit the signal for altered DNA binding has attracted increasing attention. Although the evolution of *D. radiodurans* is not fully understood, this bacterium is uniquely suited for studying bacterial adaptation to environmental stresses because of its cellular robustness. Several transcription factors have been characterized, which are critical for its growth and survival during stress conditions. Transcription factors including OxrR and PerR are important for the antioxidation of *D. radiodurans*, while DtxR and Mur appear to be involved in maintaining intracellular metal ion homeostasis (32, 33). It is worth noting that the exceptionally high intracellular Mn concentration (mM range) correlates with the extreme radio-resistance of this bacterium (34). Recent studies revealed sophisticated regulation of RDRM genes. Derepression of transcription factor DdrO bound to promoters could be activated by physical interactions between the corresponding PprI protease and single-stranded DNA (26).

The overall structure of DdrI without cAMP resembles an activated CRP-cAMP conformation, similar to active ecCRP-cAMP and mtCRP-cAMP structures. According to 16S rRNA sequences, *D. radiodurans* and *T. thermophilus* belong to the same phylum (the *Deinococcus-Thermus* phylum); therefore, it is not surprising that DdrI showed the highest structural similarity with SdrP, a CRP family protein characterized in *T. thermophilus*. Indeed, stress-related proteins of *Deinococcales* usually share structural commonalities in some ways with their *Thermales* homologs, which were frequently used as the starting search model during the structure determinations of *Deinococcus* proteins (35–37). Interestingly, SdrP was speculated to be active independent of any effector molecule including the cAMP (38). Given the distinct evolution of *Deinococcus-Thermus*, CRP family proteins from this phylum may have evolved differently from those of cAMP-dependent CRPs (e.g., ecCRP). This could also be due to different physiological and metabolic properties between *D. radiodurans* and *E. coli*, which correlated with the partial compensation for the *ddrI*-knockout phenotype by ecCRP (29). In contrast to the preferential utilization of glucose over other carbon sources in *E. coli*, *D. radiodurans* uses amino acids as the primary energy source, with fructose, pyruvate, and lactate being preferred over glucose (39). In addition, classic adenylate cyclase responsible for cAMP synthesis has not been characterized in *D. radiodurans*, although an altered cAMP concentration after radiation has been reported (40).

DdrI appears to use an alternative strategy that does not require cAMP binding (Fig. 4). Given the amphiphilic behavior of both Tyr113^n^ and adenine, residue interactions (Tyr113^n^-Arg55-Glu65) evolved in DdrI *per se* effectively mimic the cAMP binding, which is consistent with its DNA binding capability in the absence of cAMP (Fig. 3D and 4A). This is also consistent with the amino acid composition for C-helices dimerization (Fig. 1 and 2D). The C-helix of ecCRP comprises repeated hydrophobic residues except at the position of Thr127-Ser128^n^ (the TS motif). The binding of cAMP to ecCRP brings the two C-helices closer together, overcoming the interruption at the TS position and resulting in a coil-to-helix transition after the TS motif. However, in DdrI, the equivalent residue (Tyr113^n^) is anchored by Arg55, leading to an intrinsically fully folded C-helix. Indeed, hydrophobic substitutions at the TS motif (CRP* mutants) result in high CRP activities even in the absence of cAMP (41). A second factor contributing to the activated DdrI dimer without cAMP may be the varying secondary elements around the C-helix. Compared with ecCRP, DdrI has a shortened α2 helix before the C-helix and an elongated β5 strand interacting with the C-terminus of the C-helix (Fig. 2B). Furthermore, DdrI contains an additional α7 helix packed behind the D-helix (Fig. 2C). According to nuclear magnetic resonance (NMR) analysis, the D-helix undergoes a remarkable motion to form extensive interactions with the C-helix during the cAMP-mediated allosteric transition (31). Thus, these interactions may enhance the coiled-coil dimer interface formed by two C-helices, which facilitate the overall conformation of DdrI in its active CRP form.

Given the energy-unfavored *syn*-conformation and significantly lower CRP-binding affinity in the millimolar range, whether the second cAMP binding site exists under physiological conditions remains enigmatic. Nevertheless, given its location (between the DBD and NTD) and cAMP interactions with both protein (HTH motif and β4-β5 loop) and DNA, the *syn*-cAMP binding site is believed to play a biological role in the regulation of CRP activities. In the case of DdrI, all the residues, except Glu167, involved in *syn*-cAMP-ecCRP interactions are not conserved. Interestingly, among these varied *syn*-cAMP-interacting residues, high-frequency mutations at Gln170 of ecCRP (equivalent to Asp156 of DdrI) were observed in evolution experiments under a cAMP-deficient background (42). Moreover, DdrI possesses a much lower calculated isoelectric point and more negatively charged *syn*-cAMP binding site compared to those of ecCRP, featuring an altered triple-aspartate motif in the β4-β5 loop (Fig. 5). Despite their chemical similarity, previous studies have hinted at functional disparities between aspartates and glutamates. Compared with glutamate, aspartate exhibits higher conservation and frequently functions as a helix N-capping residue (43, 44), thereby accounting for the elongated β5 strand observed in DdrI (Fig. 5). Furthermore, aspartate demonstrates a proclivity to form more stable complexes with divalent cations, including Mn^2+^, in solution than glutamate (45). Considering the exceptionally elevated intracellular Mn concentration in *D. radiodurans*, it is unsurprising that Mn^2+^ proved capable of augmenting the DNA binding affinity of DdrI (Table 1). This enhancement may facilitate either transient or stable protein-DNA interactions by mitigating the negative charge repulsions between the *syn*-cAMP binding site and the DNA phosphate backbone (Fig. 6B). Indeed, the presence of Mn^2+^ is frequently documented in the structures of *Deinococcus* proteins, playing a pivotal role in enzymatic catalysis and protein folding (46, 47). As an illustration, an Mn ion is positioned at the dimer interface of the *D. radiodurans* RNase J protein, modulating its dual activity during RNA cleavage (48).

DdrI was able to form a stable complex with consensus CRP target DNA (Fig. 3D), which is consistent with previous structural, genetic, and *in silico* studies (10, 11). On the other hand, DdrI also interacted with a variant of this sequence containing an RDRM-mimic in the presence of Mn^2+^, but with a much lower binding affinity *in vitro* (Fig. S2B). Together with the increased intracellular DdrB protein (RDRM regulon) in the *ddrI* knockout strain and the partial overlap between the putative DdrI binding sequence and RDRM, our results further confirmed potential cross-talk between DdrI and DdrO. Other factors may also contribute to such cross-talk that one RDRM could be regulated by these two transcription factors: (i) Two residues equivalent to Gly184 and Arg185 of ecCRP are not strictly conserved across different CRPs. In contrast to the unchanged activity upon conservative substitution of R185K, Gly184 is important for both DNA binding and RNA polymerase (RNAP) recruitment by ecCRP (49). Therefore, variations in these two residues in DdrI may have an impact on RNAP interacting with RDRM. (ii) Interactions between activating regions of CRP and RNAP are required for its cellular function. Our DdrI structure superimposed well onto TAP (another CRP homolog in *T. thermophilus*) in the intact bacterial class II transcription activation complex (PDB ID: 5I2D) (50). TAP also contains an additional helix equivalent to α7 of DdrI, which interacts with the RNAP α subunit C-terminal domain. Notably, cAMP was not required for this complex formation, which confirms the active conformation of DdrI solved in the current study. Given the interactions between α7 and the D-helix, this may explain a prerecruitment mechanism that differs from ecCRP recruitment (51); CRPs with an additional helix may bind to RNAP prior to DNA. Moreover, the mechanism of RNAP recruitment after DdrO cleavage remains unclear. Based on the possible RDRM binding and RNAP interaction of DdrI, DdrI may function as a mediator for RDRM activation after DNA damage. (iii) Given the low binding affinity of *syn*-cAMP, other effector molecules, such as cGMP, could also be involved in DdrI regulation. It should be noted that the *relA* function is normal in *D. radiodurans*, and it is induced under growth conditions with limited amino acids (52), the most preferred carbon source for this organism. Thus, ppGpp synthesized by RelA is also a candidate DdrI effector. (iv) Single-molecule analyses have revealed cooperative binding of transcription factors to DNA, which is highly dynamic (53). The highly condensed genome of *D. radiodurans* is expected to contribute to its robustness with less damage from environmental stresses. Factors such as local DNA structures or cooperative/competitive binding between multiple transcription factors may also regulate RDRM gene expression.

To adapt to their environment, bacteria have evolved diverse transcription factors that participate in the global regulation of gene expression. On the other hand, the same transcription factors in different bacteria have also evolved to help them adapt to specific environments according to their characteristics. The DdrI protein in this study is one example, exhibiting different structural and biochemical properties from classical CRPs. Given that the characteristics of ecCRP are likely to have evolved from a simple DNA-binding protein with limited specificity (54), elucidating the cAMP-independent regulatory mechanism of this protein and its possible cross-talk with other transcription factors, such as DdrO, would provide valuable information for better understanding the environmental adaptability of the extreme microorganism *D. radiodurans*.

## MATERIALS AND METHODS

### Bacterial strains, plasmids, and growth conditions

*D. radiodurans* strains (ATCC 13939) were grown at 30°C in TGY broth (0.5% tryptone, 0.3% yeast extract, and 0.1% glucose) or on TGY plates with 1.5% (wt/vol) agar powder. *E. coli strains* were cultivated in LB broth (1% tryptone, 0.5% yeast extract, and 1% NaCl) or on LB plates with 1.5% (wt/vol) agar at 37°C. The full-length *ddrI* gene (DR0997, GenBank accession number A2G07_08515) was amplified by PCR from *D. radiodurans* genomic DNA and cloned into the NdeI and BamHI sites of the pET28-HMT vector (26). Site-directed mutation of the DdrI sequence (D156Q) was generated using a QuickChange site-directed mutagenesis kit (Stratagene) as previously described (37). All the strains and plasmids are listed in Table S2. All the primers and DNA substrates are listed in Table S3.

### Protein expression and purification

The DdrI expression strains were grown in LB broth to an optical density at 600 nm of 0.6–0.8, followed by adding isopropyl-β-d-thiogalactopyranoside (IPTG) at a final concentration of 0.2 mM. The cells were harvested by centrifugation and resuspended in lysis buffer (20 mM HEPES pH 7.5, 500 mM KCl, 5% glycerol, 1 mM PMSF, and 20 µg/mL lysozyme) and lysed by sonication. After centrifugation, the supernatant was purified by an AKTA Purifier system: The supernatant was loaded onto a HisTrap HP column (GE Healthcare) pre-equilibrated with buffer A (20 mM HEPES pH 7.5, 500 mM KCl, 1 mM TCEP, and 5% glycerol) and finally eluted with 200 mM imidazole. After TEV protease cleavage, the samples were desalted by the HiPrep 26/10 Desalting column (GE Healthcare) with buffer B (20 mM HEPES pH 7.5, 100 mM KCl, 1 mM TCEP, and 5% glycerol). The proteins were subsequently loaded onto HiTrap Q HP column (GE Healthcare) pre-equilibrated with buffer B and eluted with a linear gradient from 100 to 500 mM KCl. The proteins were finally purified by Superdex 75 Increase 10/300 Gl column (GE Healthcare) with buffer C (20 mM HEPES pH 7.5, 100 mM KCl, and 1 mM TCEP). Fractions containing the purified proteins were pooled, concentrated, and stored at −80°C. D156Q was expressed and purified by similar methods to those described for wild-type DdrI.

### Crystallization and structure determination

DdrI crystals (~15 mg/mL) were grown using the drop vapor diffusion method at 289K over wells containing 0.1 M Bis-Tris pH 6.5, 2.5 M KCl. Cryocooling was achieved by stepwise soaking the crystals in a reservoir solution containing 10%, 20%, and 30% (wt/vol) glycerol. The diffraction intensities were recorded at Shanghai Synchrotron Radiation Facility (Shanghai, China) and were integrated and scaled using the XDS suite (55). The structure was determined by molecular replacement method using SdrP (PDB ID: 2ZCW) as the search model (56). Structures were refined using PHENIX (57) and interspersed with manual model building using COOT (58). Later stages of refinement employed TLS group anisotropic B-factor refinement. All the residues are in the most favorable and allowed regions of the Ramachandran plot. The final model comprises residues 12–202. The statistics for data collection and reﬁnement are listed in Table S1.

### Electrophoretic mobility shift assays

Standard EMSA reaction mixture consisted of 20 mM HEPES pH 7.5, 100 mM NaCl, 20 µg/mL bovine serum albumin (BSA), 1 mM TCEP, 100 nM 5ʹ-Cy5-labeled DNA, and 1–3 μM DdrI. The reactions could contain 10 µM Mn^2+^ and 100 µM cAMP if necessary. The reaction mixture was incubated at 30°C for 30 min, loaded onto 20% (wt/vol) polyacrylamide gels, and run for 80 min at 180 V in 1 × Tris-borate buffer at 4°C (ice-water bath). The gel was then photographed using a Typhoon 9500 (GE Healthcare).

### MST assays

To determine the DNA binding affinity constants, 20 nM Cy5-labeled DNA was incubated with a dilution series of DdrI (0.305 nM to 10 µM) in an MST buffer (20 mM HEPES pH 7.5, 100 mM KCl, and 0.05% Tween 20) for 30 min at room temperature. The reactions could contain 10 µM Mn^2+^ and 100 µM cAMP if necessary. The MST measurements were performed using the Monolith NT.115 instrument (NanoTemper Technologies, München, Germany). For cAMP binding affinity measurements, N-terminal-eGFP fused DdrI was incubated with a dilution series of cAMP (1.2 µM to 2.5 mM) in MST buffer. Data were analyzed using MO.Affinity Analysis software and GraphPad Prism 8. Binding curves represent the change in normalized fluorescence (fraction bound or Fnorm), and *K*_d_ values were calculated using the K_d_ Fit Model. All experimental data were obtained from three independent replicates (Table 1).

### Molecular dynamics simulations

We designed our computational model by superimposing our resolved DdrI dimer structure onto the ecCRP-DNA complex (PDB ID: 2CGP), selectively retaining the cAMP molecules in the second binding site and mutating the DNA sequence into a representative RDRM-mimic (AAATGTTATGTCAAAAACATG). For DdrI protein, the AMBER14SB force field was utilized, while the DNA was modeled using the OL21 force field. The cAMP molecules were parametrized with the Generalized Amber Force Field and assigned AM1-BCC charges. The system was solvated with TIP3P water in a 1-nm buffered cubic box and neutralized with 150 mM NaCl with tleap in AmberTools23. Quantities of ions were calculated by SLTCAP server. A cutoff of 1 nm is applied for non-bonded interactions. The reference temperature and pressure were 310.0K and 1.0 bar, respectively.

Following a 10,000-step steepest descent energy minimization and subsequent 0.5 ns NVT and 0.5 ns NPT equilibrations, we run molecular dynamics simulations for 1,000 ns each round. In the first round, excessive Mn^2+^ was added to probe the most probable binding regions, employing the Li-Merz 12-6-4 LJ potential. Four independent MD simulations were performed using Amber22 software. In the second round of simulations, nonspecifically bound Mn^2+^ ions were removed from the last frame of each trajectory in the first round and further equilibrated the system by running two independent simulations each. The remaining Mn^2+^ ions were parameterized using the Li-Merz 12-6 nonbonded model so that the interaction between Mn^2+^ and other molecules was slightly weakened to confirm the stability of Mn^2+^ binding. Simulations were performed using Gromacs 2023.2. The coordinates were saved every 0.5 ns. The trajectory visualization and clustering analysis were conducted using VMD 1.9.4a57. The MDAnalysis Python package was used for contact analysis and distance calculation. Effective contact with Mn^2+^ ions is defined when O/N/S atoms from at least three protein residues, nucleotides, or the cAMP molecule are within 2.5 Å distance around the Mn^2+^ ion.

## Data Availability

The coordinate and structure factor have been deposited to the Protein Data Bank with accession code 8YZ7.

## SUPPLEMENTAL MATERIAL

Supplemental figures and tables mbio.01144-24-s0001.doc Fig. S1 to S2 Tables S1 to S3.

ASM does not own the copyrights to Supplemental Material that may be linked to, or accessed through, an article. The authors have granted ASM a non-exclusive, world-wide license to publish the Supplemental Material files. Please contact the corresponding author directly for reuse.
