# Supplementary material for: cAMP-independent DNA binding of the CRP family protein DdrI from Deinococcus radiodurans
Source: mBio. 2024 Jun 25;15(7):e01144-24. doi: 10.1128/mbio.01144-24 (PMC11253593; doi:10.1128/mbio.01144-24)
Supplement: Supplemental figures and tables — Fig. S1 to S2 Tables S1 to S3. [file mbio.01144-24-s0001.doc]

**cAMP-independent DNA binding of the CRP family protein DdrI from *Deinococcus radiodurans***

Yudong Wang^1^, Jing Hu^1^, Xufan Gao^2^, Yuchen Cao^1^, Shumai Ye^1^, Cheng Chen^1^, Liangyan Wang^1^, Hong Xu^1^, Miao Guo^1^, Dong Zhang^1,2^, Ruhong Zhou^1,2,3,4^, Yuejin Hua^1^ & Ye Zhao^1*^

^1^. MOE Key Laboratory of Biosystems Homeostasis & Protection, Institute of Biophysics, College of Life Sciences, Zhejiang University, China

^2^. Institute of Quantitative Biology, College of Life Sciences, Zhejiang University, Hangzhou, China

^3^. Shanghai Institute for Advanced Study, Zhejiang University, 799 Dangui Road, Shanghai, China

^4^. Department of Chemistry, Columbia University, New York, United States

* To whom correspondence should be addressed. Tel: 86-571-86971279; fax: 86-571-86971279; email: Z. Y. (yezhao@zju.edu.cn)

**Keywords:** transcription factor; DNA binding; dimer; cAMP; allosteric effect; microscale thermophoresis

Running title: cAMP-independent CRP family protein DdrI

**Table S1: Data collection and refinement statistics**

|  | DdrI |
| --- | --- |
| PDB ID | 8YZ7 |
| **Data collection** |  |
| Space group | P 3_2_21 |
| Cell dimensions |  |
| a, b, c (Å) | 87.17 87.17 61.64  90 90 120 |
|  |  |
| Wavelength (Å) | 0.9792 |
| Resolution (Å) | 30-2.0 |
| Rsym (%) | 7.3 (61.3) |
| I/σI | 23.7 (5.4) |
| Completeness (%) | 100.0 (100.0) |
| Redundancy | 19.7 (20.2) |
|  |  |
| **Refinement** |  |
| Resolution (Å) | 30-2.0 |
| No. reflections | 18610 |
| Rwork/ Rfree | 20.2/22.6 |
| No. atoms |  |
| Protein | 1452 |
| ion | 3 |
| B-factors |  |
| Protein | 52.1 |
| ion | 74.6 |
| R.m.s deviations |  |
| Bond lengths (Å) | 0.005 |
| Bond angles (º) | 0.738 |
|  |  |
| Ramachandran statistics |  |
| Favored (%) | 98.4 |
| Allowed (%) | 1.6 |
| Outliers (%) | 0 |

Statistics for the highest-resolution shell are shown in parentheses.

**Table S2: Strains and plasmids**

| **Strain and plasmid** | **Description** | **Source** |
| --- | --- | --- |
| **Strains** |  |  |
| ***E. coli*** |  |  |
| Trans5α | *E. coli* cloning strain | Transgen |
| BL21 (DE3) | *E. coli* expression strain | Transgen |
| BL21-HMT-DdrI | *E. coli* expression strain for DdrI protein with removable tag | This study |
| BL21-HMT-eGFP-DdrI | *E. coli* expression strain for DdrI protein with eGFP tag | This study |
| BL21-HMT-DdrI D156Q | *E. coli* expression strain for DdrI (D156Q) | This study |
| ***D. radiodurans*** |  |  |
| wild-type strain | ATCC 13939 | Lab stock |
| **Plasmids** |  |  |
| pMD18-T vector | For TA cloning | Lab stock |
| pET28-HMT | Modified from pET28a containing 6×His-tag, maltose binding protein and TEV protease site | Lab stock |
| pET28-HMT-*ddrI* | As pET28a-HMT but ligated with *ddrI* | This study |
| pET28-HMT-*ddrI D156Q* | As pET28a-HMT but ligated with *ddrI D156Q* | This study |
| pET28-HMT-*egfp*-*ddrI* | As pET28a-HMT but ligated with *egfp*-*ddrI* | This study |

**Table S3: Primers and DNA**

|  | **Sequence (5′→3′)** | **Label** |
| --- | --- | --- |
| **EMSA assays** | |  |
| 18 bp target DNA | A**TGTGA**CTTCAC**TCACA**C | 5ʹ-Cy5 |
| 24 bp target DNA | ACTA**TGTGA**CTTCAC**TCACA**CTGT | 5ʹ-Cy5 |
| 26 bp target DNA | TCCTA**TGTGA**CTTCAC**TCACA**CTGTC | 5ʹ-Cy5 |
| 28 bp target DNA | ATACTA**TGTGA**CTTCAC**TCACAC**TGTCG | 5ʹ-Cy5 |
| **MST assays** |  |  |
| 28 bp target DNA | ATACTA**TGTGA**CTTCAC**TCACA**CTGTCG | 5ʹ-Cy5 |
| 28 bp *ddrB*-mimic | ATACTA**TGTTA**TGTTAT**TTACG**CTGTCG | 5ʹ-Cy5 |
| 28 bp *ddrC*-mimic | ATACTA**TGTTA**TGTCAA**AAACA**CTGTCG | 5ʹ-Cy5 |
| **Clone and site mutation** | |  |
| *ddrI* F | gaaaacctgtattttcagggc**catATG**ACCCAGACCCAGACTGCT | NdeI |
| *ddrI* R | acggagctcgaattc**ggatcc**TTAGGCCGCCGCGATGGT | BamHI |
| *ddrI* D156Q F | CACCCAC**CAG**GAACTCGCCGCCGC |  |
| *ddrI* D156Q R | CGAGTTC**CTG**GTGGGTGGCGTAGATCATCGTT |  |

**Supplementary Figures**

**
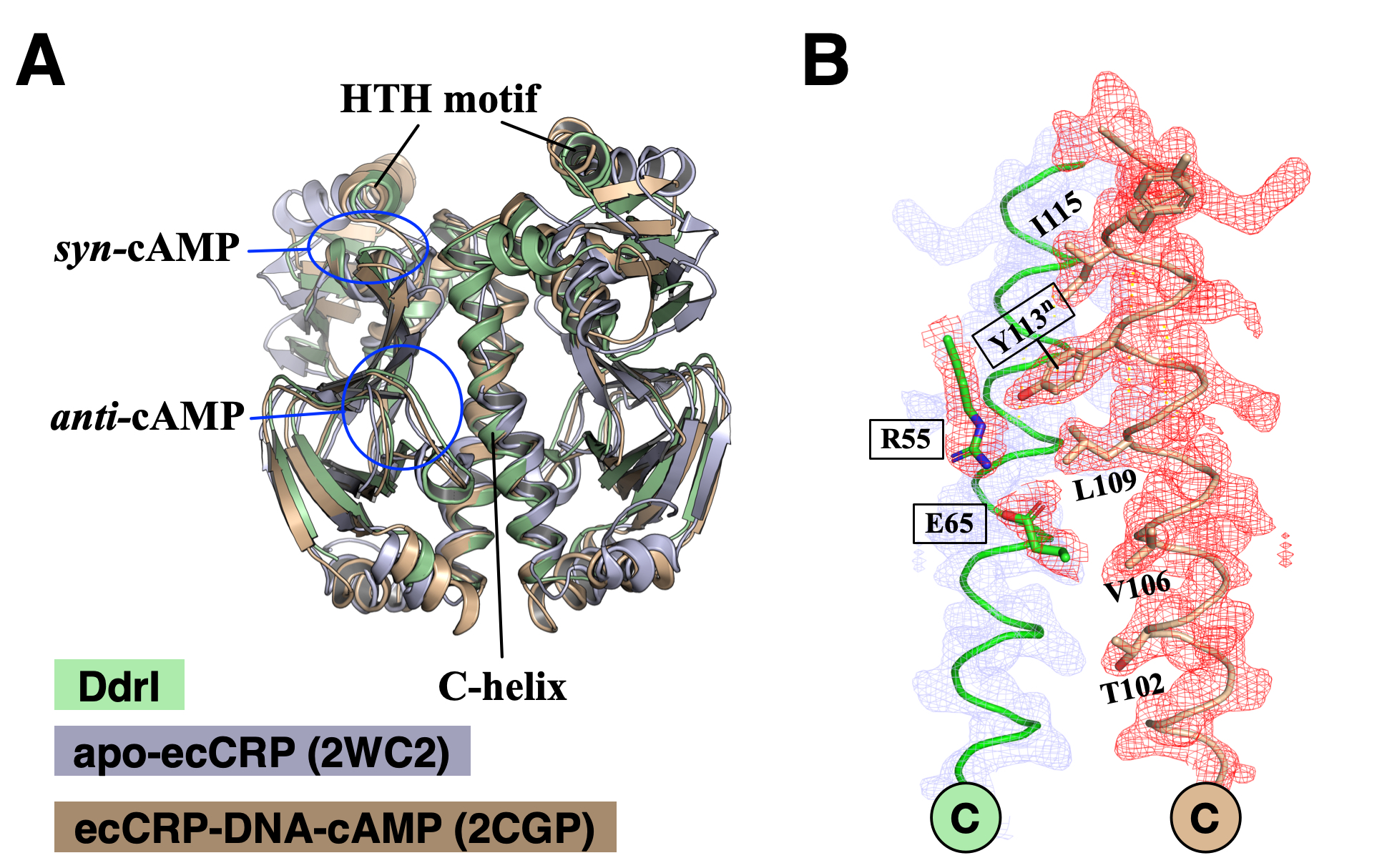
**

**Fig. S1. Overall structure of DdrI. (A)** Superposition of DdrI dimer (green) with apo-ecCRP (grey, 2WC2) and ecCRP-DNA-cAMP (brown, 2CGP). The C-helix and HTH motif are labeled and two cAMP binding sites within one protomer are highlighted with blue circles. **(B)** The electron densities of fully folded C-helices and Tyr113^n^-Arg55-Glu65 are shown in red with the refined 2Fo-Fc map contoured at 1𝜎.


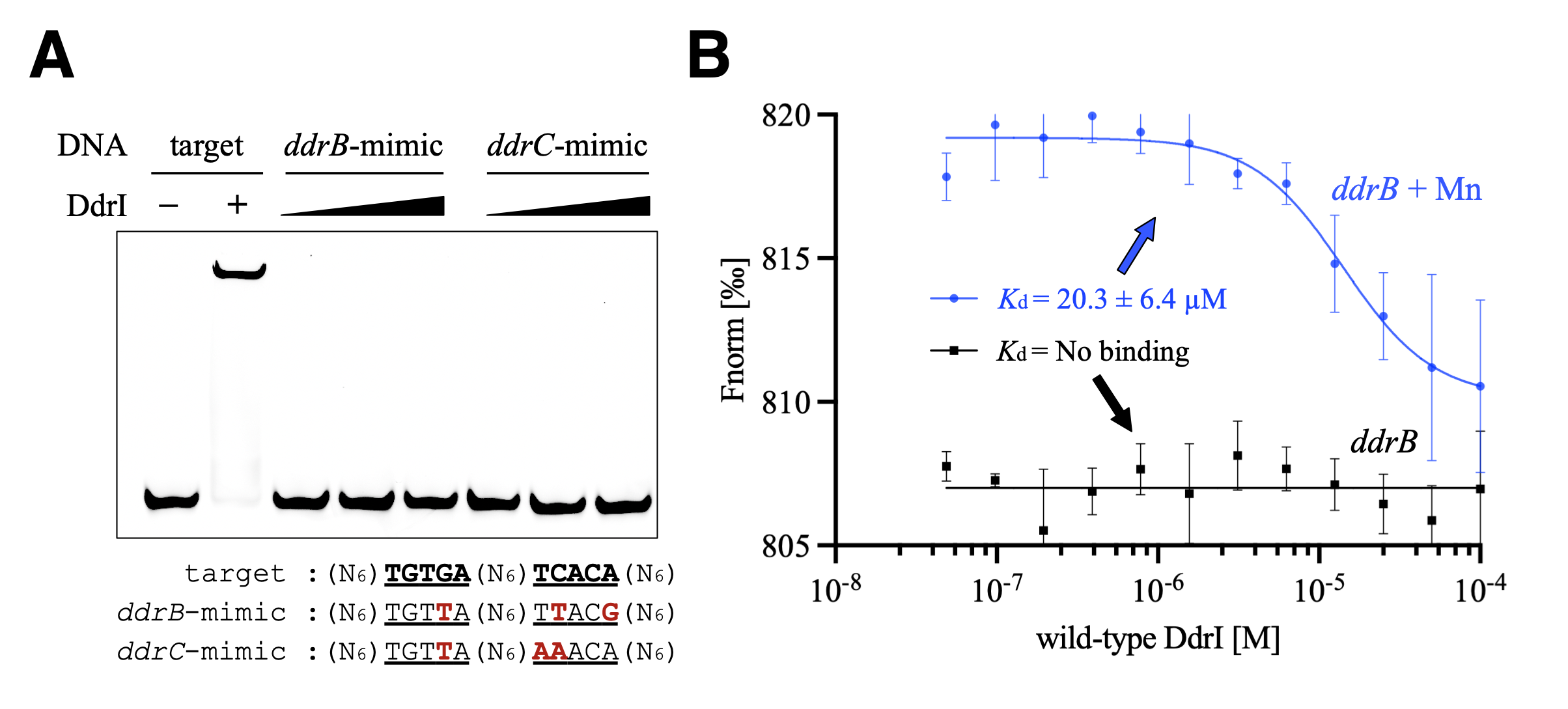


**Fig. S2. DNA binding capabilities of DdrI. (A)** EMSA shows that DdrI cannot form a stable complex with the RDRM-mimic DNA. 5ʹ-Cy5 labeled target DNA containing 100 nM 28 bp target DNA (target) or RDRM-mimicking sequence (*ddrB* and *ddrC*) was incubated with 1, 2, and 3 µM DdrI protein at 30℃ for 30 min. **(B)** *ddrB*-mimicking DNA binding affinities of DdrI. Target DNA (28bp) was labeled with 5ʹ-Cy5 and maintained at a constant concentration of 20 nM in MST buffer, followed by protein titration from 24.4 nM to 100 µM. Binding curves represent the change in normalized fluorescence (Fnorm[‰]), and the *Kd* values were calculated using the K_d_ Fit Model.
